# Contemporary use and outcome of Cabrol shunt in type A aortic dissection surgery: insight from China 5A study

**DOI:** 10.1136/openhrt-2023-002465

**Published:** 2023-12-09

**Authors:** Hong Liu, Bing-qi Sun, Si-chong Qian, Ming-yu Sun, Yong-feng Shao, Yi Ding, Hui Li, Hong-jia Zhang

**Affiliations:** 1 Department of Cardiovascular Surgery, the First Affiliated Hospital of Nanjing Medical University, Nanjing, China; 2 Department of Cardiovascular Surgery, Teda International Cardiovascular Hospital, Peking Union Medical College & Chinese Academy of Medical Sciences, Tianjin, China; 3 Department of Cardiovascular Surgery, Beijing Anzhen Hospital, Capital Medical University, Beijing, China

**Keywords:** Aneurysm, Dissecting, Cardiac Surgical Procedures, Aortic Aneurysm

## Abstract

**Objective:**

Cabrol shunt has been introduced for surgical repair of type A aortic dissection (TAAD) without robust evidence supporting its routine preventive use.

**Methods:**

Adult patients with TAAD from China 5A study were included if surgically repaired between 2016 and 2022. Primary outcome was operative mortality according to Society of Thoracic Surgeons criterion. Overall, we compared clinical outcomes in patients with and without Cabrol shunt, and subgroup analysis were further examined between Cabrol shunt and outcome among patients with or without root replacement.

**Results:**

3283 patients were finally identified for analysis, with median age of 51 (IQR 41–59) years, 2389 men, and 2201 treated with Cabrol shunt technique. Cabrol shunt-treated patients were more severely ill before surgery than those without Cabrol shunt. Overall, the rate of operative mortality was 6.6% (146/2201 in Cabrol shunt group and 71/1082 in non-Cabrol shunt group), with no association between Cabrol shunt and operative mortality (OR 1.012 (95% CI 0.754 to 1.357); p=0.938). Stratified by root replacement, Cabrol shunt was associated with similar risk of operative mortality either in patients without root replacement (OR 1.054 (0.747 to 1.487); p=0.764) or in patients with root replacement (OR 1.194 (0.563 to 2.536); p=0.644) (P interaction=0.765). Results were similar in multiple sensitivity analysis.

**Conclusion:**

Cabrol shunt was not associated with either a greatly lowered or an increased risk of operative mortality, regardless of aortic root replacement. Our study did not support the use of Cabrol shunt as a routine preventive strategy in the treatment of TAAD.

**Trial registration number:**

NCT04398992

**WHAT IS ALREADY KNOWN ON THIS TOPIC**

- Cabrol shunt is a technique used for uncontrolled bleeding during graft replacement of ascending aortic and aortic root within the aneurysmal sac.
- Cabrol shunt has been used as a technique to control bleeding in surgical repair of type A aortic dissection, especially in cases of excessive or uncontrolled bleeding, coagulation disorder and reoperation.

**WHAT THIS STUDY ADDS**

- Cabrol shunt was not associated with either a greatly lowered or an increased risk of operative mortality.
- Cabrol shunt was associated with similar risk of operative mortality either in patients without root replacement or in patients with root replacement, without interaction effect.

**HOW THIS STUDY MIGHT AFFECT RESEARCH, PRACTICE OR POLICY**

- Cabrol shunt as a routine preventive strategy should be used with caution in the treatment of type A aortic dissection.

## Introduction

Although surgical techniques and haemostatic agents have been significantly improved, suture lines bleeding is still challenging after surgical repair of acute type A aortic dissection (TAAD), mainly due to the suture line location in the dissected aortic segment, impaired blood coagulability and prolonged cardiopulmonary bypass time runs.^1–4^ Cabrol shunt or Cabrol fistula, also known as a ‘perigraft-to-right atrial shunt’, is a technique used for uncontrolled bleeding during graft replacement of ascending aortic and aortic root within the aneurysmal sac (inclusion technique).^4^ A perigraft to right atrial fistula is created by wrapping the neo-aorta using the native aneurysmal aortic wall and shunting the aneurysm sac to the right atrium for autotransfusion of the bleeding into the circulation, which decompresses the perigraft space to avoid external graft compression or tension on anastomotic suture lines, consequently reducing the risk of complications such as pseudoaneurysm of the coronary ostia or of the distal aortic suture.^5–7^ Therefore, Cabrol shunt has been proposed as an effective technique to control bleeding in surgical repair of TAAD, especially in cases of excessive or uncontrolled bleeding, coagulation disorder and reoperation. In addition to the classic Cabrol shunt, several modified Cabrol shunt techniques subsequently were described and applied in either open or inclusion repair of aortic dissection for the prophylactic, routine or remedial purpose, with satisfactory haemostasis results.^8 9^ However, Cabrol shunt usually requires preserving its naïve aortic wall, which limited precise anatomic reconstruction as well as heart failure and severe pulmonary hypertension if the shunt did not close in the short-term.^10 11^

Currently, Cabrol shunt and its modifications remains used in part of Chinese aortic centres when dealing with surgical repair of TAAD. However, surgical management of aortic aneurysms/dissections has changed in the last decades, so a detailed knowledge is mandatory for adequate interpretation of current use and outcomes of Cabrol shunt techniques. In this multicentre observational study from China, we investigate the perioperative outcomes of Cabrol shunt and to determine whether Cabrol Shunt can be a routinely preventive procedure in TAAD surgery.

## Methods

### Study design and ethical approval

The China Additive Anti-inflammatory Action for Aortopathy and Arteriopathy (5A) registry study is an ongoing prospective, multicentre cohort registry designed to collect data on clinical backgrounds and outcomes of patients hospitalised for aortic dissection since January 2016. This present study was conducted in accord with the Declaration of Helsinki and registered in ClinicalTrials.gov. Patient written consent for the publication of the study data was waived due to this retrospectively observational study. Patient selection, data collection and data analysis were performed in accordance with STROBE (Strengthening the Reporting of Observational Studies in Epidemiology) guidelines and TRIPOD guidelines (Transparent Reporting of a multivariable prediction model for Individual Prognosis or Diagnosis).^12 13^

### Patient selection

**Figure 1:**
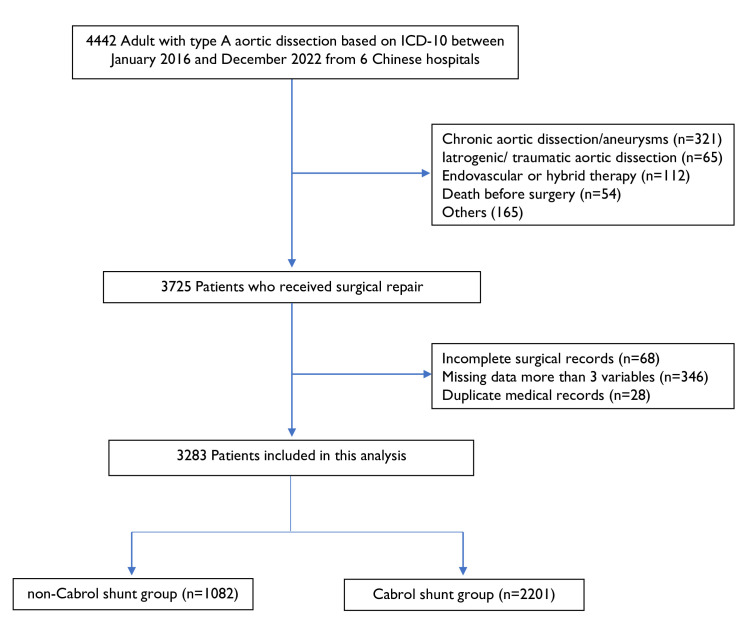
Patient selection flow chart. ICD-10, International Classification of Diseases 10th Edition; TAAD, type A aortic dissection.

From January 2016 to December 2022, consecutive patients with acute type A aortic dissection (ATAAD) hospitalised through the emergency department at participating hospitals were retrospectively identified from six hospitals of China 5A study at their first admission and then followed after discharge (figure 1). ATAAD was defined as any dissection involving the ascending aorta on contrast-enhanced CT, within 14 days of symptom onset. Preoperative diagnosis was made on contrast-enhanced CT angiography. Patients 18 years of age or older were included if they underwent aortic surgery within 14 days from symptom onset to hospital arrival. Key criteria for exclusion included type B aortic dissection, recurrent aortic dissection, traumatic aortic dissection and iatrogenic aortic dissection.

### Data collection

Patient information obtained included demographic data, medical history and risk factors, baseline characteristics, surgical procedures, pharmacotherapeutics, critical care and discharge. The detailed definitions and measures of cigarette smoking, alcohol consumption, body mass index, hypertension, diabetes mellitus, arrhythmia and malperfusion was previous in our recent report.^14^ All central laboratories at participating sites have been recognised and certified by the China National Accreditation Service for Conformity Assessment of Laboratory.

### Surgical procedure

Surgical treatment of TAAD were mainly approached by two basic methods: placement of an interposition graft with resection of the dissected tissue, and the inclusion graft technique in which the remnant of the native aorta is wrapped around the graft. Graft inclusion was performed according to the technique described by Bentall.^15^ The ascending aorta was reserved and was incised longitudinally, and Dacron graft was anastomosed inside and into the true lumen of the aorta. The remnant of the native aorta is wrapped around the graft, and a surgical fistula (Cabrol shunt) from the periaortic space to the right atrium was created to decompress the perigraft space for control of bleeding (autotransfusion). Several modifications of Cabrol shunt have been performed in surgical repair of TAAD, in which a perigraft compartment is created by the placement of an autologous pericardium or a bovine pericardial patch that is wrapped around the graft. The decision to perform Cabrol shunt was in a non-randomised fashion and at the surgeon’s discretion for the purpose of prophylactic, routine or remedial use.

### Outcome

The primary outcome was operative mortality, defined as any death, regardless of cause, occurring within 30 days after surgery in or out of the hospital and after 30 days during the same hospitalisation subsequent to the operation according to the Society of Thoracic Surgeons criteria.^16^ Other outcomes were 30-day mortality, mechanical ventilation time and intensive care unit stay. Malperfusion was defined as end-organ ischaemia caused by branch vessel involvement and resulting in functional impairment.^17^ In this study, the follow-up for patients was concluded 30 days after surgery, hospital discharge or the death occurring during the same hospitalisation subsequent to the operation, which ever last.

### Statistical analysis

Logistic regression was used to estimate the association between Cabrol shunt and outcome by OR and 95% CI. This association was further evaluated with adjustment for the potential baseline, demographic, comorbidity, clinical and laboratory confounders. Besides, three propensity score (PS) methods were employed to control the imbalance from the non-randomised use of open versus inclusion technique and to alleviate the effects of confounding factors: inverse probability weighting (IPTW) analysis, GenMatch matching analysis and PS as an additional covariate.^18–20^ In particular, GenMatch is a matching technique that combines PS matching with multivariate matching, which weights the PS and the observed individual variables based on an automated search algorithm and iteratively checks the balance and directs the search to ward the best matches (those that optimise balance).^21^ We performed subgroup analysis to investigate whether there was an interaction between Cabrol shunt, aortic root replacement and operative mortality.^22^

We further calculated the number needed to treat, which is a measure that indicates how many patients on average need to be treated with Cabrol shunt to prevent an average of one patient from operative death, or the number needed to harm, which is a measure that indicates how many patients on average need to be exposed to Cabrol shunt to cause death in an average of one patient who would not otherwise die.^23^

Continuous data were presented as mean (SD) or median (IQRs) compared by t-test or Mann-Whitney test, and categorical data were reported as percentages (%) compared by χ^2^ test or Fisher exact test. P values were two-tailed, with p value<0.05 considered statistically significant unless otherwise stated. Statistical analysis was performed using R software, Stata statistical software and Python programming software.

## Results

### Patient characteristics and outcome

Three thousand two hundred and eighty-three patients were finally identified for analysis, with median age of 51 (IQR 41–59) years and 2389 men, of whom 2201 underwent Cabrol shunt technique. Patient characteristics and perioperative outcomes stratified by absence versus presence of Cabrol shunt technique were presented in table 1. Cabrol shunt-treated patients were more severely ill before surgery than those without Cabrol shunt. Overall, the rate of operative mortality was 6.6% (146/2201 in the Cabrol shunt group and 71/1082 in the non-Cabrol shunt group).

**Table 1:** Patient characteristics, operative data and outcomes

	Non-Cabrol shunt group (N=1082)	Cabrol shunt group (N=2201)	P value
Demographic profiles			
Age (years)	50.0 (39.0 to 59.0)	51.0 (42.0 to 59.0)	0.174
Female sex (%)	347 (32.1)	547 (24.9)	<0.001
Height (cm)	171.0 (165.0 to 176.0)	172.0 (166.0 to 176.0)	0.565
Weight (kg)	74.0 (65.0 to 83.0)	75.0 (65.0 to 85.0)	0.029
Body mass index (kg/m^2^)	25.3 (22.8 to 27.7)	25.4 (23.3 to 28.0)	0.006
Clinical history and risk factors			
Smoking (%)	431 (40.6)	941 (43.4)	0.122
Drinking (%)	229 (22.0)	454 (21.4)	0.689
Hypertension (%)	709 (66.1)	1625 (74.3)	<0.001
Diabetes (%)	53 (4.9)	118 (5.4)	0.575
Arrhythmias (%)	26 (2.4)	59 (2.7)	0.642
Chronic lung disease (%)	55 (5.1)	51 (2.3)	<0.001
Stroke (%)	60 (5.5)	95 (4.3)	0.121
Coronary heart disease (%)	102 (9.4)	201 (9.1)	0.781
Shock/hypotension (%)	43 (4.0)	31 (1.4)	<0.001
Antiplatelet (%)	12 (1.1)	29 (1.3)	0.503
Anticoagulants (%)	10 (1.0)	26 (1.2)	0.379
Laboratory profiles			
Leucocyte (×10^9^ /L)	8.1 (6.0 to 11.9)	8.9 (6.5 to 12.3)	<0.001
Platelet (×10^9^ /L)	201.0 (162.0 to 246.0)	187.0 (152.0 to 232.0)	<0.001
Creatine kinase-MB (ng/mL)	1.2 (0.7 to 2.2)	1.3 (0.8 to 2.5)	<0.001
Lactic dehydrogenase (μ/L)	190.0 (160.0 to 236.5)	196.0 (165.0 to 239.0)	0.226
D-dimer (ng/mL)	663.0 (202.0 to 2353.0)	808.5 (201.0 to 2401.2)	0.540
Lactic acid (mmol/L)	1.4 (1.0 to 1.9)	1.4 (1.0 to 1.9)	0.593
Alanine transaminase (μ/L)	20.0 (13.8 to 31.0)	20.0 (14.0 to 31.0)	0.783
Aspartate aminotransferase (μ/L)	20.0 (16.0 to 26.9)	21.0 (16.0 to 28.0)	0.017
Albumin (g/L)	40.3 (35.9 to 43.2)	40.0 (36.8 to 42.9)	0.137
Urea nitrogen (mmol/L)	5.7 (4.6 to 7.1)	6.1 (4.8 to 7.6)	<0.001
Haemoglobin (g/L)	139.0 (126.0 to 150.0)	138.0 (126.0 to 149.0)	0.706
Creatinine (μmoI/L)	73.7 (59.9 to 87.4)	75.6 (64.1 to 92.6)	<0.001
International normalised ratio	1.1 (1.0 to 1.1)	1.1 (1.0 to 1.2)	0.035
APTT (s)	30.6 (28.1 to 33.3)	30.2 (28.0 to 32.7)	0.058
PH	7.4 (7.4 to 7.4)	7.4 (7.4 to 7.4)	<0.001
Arterial carbon dioxide tension (mm Hg)	36.3 (33.2 to 40.0)	34.8 (31.8 to 37.8)	<0.001
Base excess (mmol/L)	−0.2 (−1.7 to 1.4)	−0.7 (−2.1 to 0.8)	<0.001
Glucose (mmol/L)	5.7 (4.9 to 7.2)	6.3 (5.1 to 7.6)	<0.001
Dissection-related characteristics			
Malperfusion* (%)	153 (16.6)	823 (37.4)	<0.001
Pericardial tamponade (%)	68 (6.5)	219 (10.7)	<0.001
Aortic regurgitation (%)			<0.001
Mild	333 (31.7)	665 (32.5)	
Moderate	44 (4.2)	355 (17.4)	
Severe	72 (6.9)	542 (26.5)	
Pericardial effusion (%)			<0.001
Mild	71 (6.6)	239 (10.9)	
Moderate	9 (0.8)	50 (2.3)	
Severe	4 (0.4)	26 (1.2)	
Procedure characteristics			
Root procedures			<0.001
Aortic valve replacement (%)	47 (4.3)	60 (2.7)	
Root replacement (%)	154 (14.2)	1107 (50.3)	<0.001
Bentall (%)	105 (9.7)	1063 (48.3)	
David (%)	41 (3.8)	11 (0.5)	
Cabrol (%)	8 (0.7)	33 (1.5)	
Arch replacement (%)	370 (34.2)	1673 (76.0)	<0.001
Hemiarch replacement	67 (6.2)	315 (14.3)	
Total arch replacement	303 (28.0)	1358 (61.7)	
Concomitant CABG (%)	56 (5.2)	205 (9.3)	<0.001
Concomitant valve surgery (%)	33 (3.0)	118 (5.4)	0.003
Total arch replacement+FET implantation (%)	294 (27.2)	1340 (60.9)	<0.001
Cardiopulmonary bypass time (min)	152.0 (125.0 to 185.0)	181.0 (145.0 to 214.0)	<0.001
Aortic cross-clamp time (min)	91.0 (66.0 to 131.0)	102.0 (83.0 to 126.0)	<0.001
Circulatory arrest time (min)	22.0 (17.0 to 28.0)	23.0 (18.0 to 30.0)	0.003
Perioperative outcomes			
Operative mortality (%)	71 (6.6)	146 (6.6)	0.938
30-day mortality (%)	55 (5.1)	134 (6.1)	0.245
ICU stay (hours)	20.0 (16.0 to 40.0)	36.7 (19.2 to 79.0)	<0.001
Mechanical ventilation time (hrs)	17.2 (14.0 to 26.3)	19.0 (14.8 to 42.0)	<0.001
Hospital stay (days)	18.0 (14.0 to 26.0)	19.0 (13.0 to 25.0)	0.045

Data are n (%) or median (IQR).

*Defined as one of the following conditions: coronary malperfusion, renal malperfusion, cardiogenic shock (cardiac tamponade, low cardiac output), cerebral perfusion (cerebrovascular accident in the previous 24 hours) and any pulse deficit/limb ischaemia.

APTT, activated partial thromboplastin time; CABG, coronary artery bypass grafting; FET, frozen elephant trunk; ICU, intensive care unit.

### Association of Cabrol shunt with outcome

Overall, with no association between Cabrol shunt and operative mortality (OR 1.012 (95% CI 0.754 to 1.357); p=0.938) (table 2). After adjustment for age, sex, body mass index, cardiovascular risk factors and laboratory profiles, the risk remained similar in multivariable analysis (OR 0.990 (95% CI 0.682 to 1.437); p=0.957), PS adjustment (OR 1.020 (95% CI 0.734 to 1.418), p=0.904), PS matching (OR 0.663 (95% CI 0.361 to 1.219); p=0.186) and IPTW analysis (OR 0.968 (95% CI 0.644 to 1.454); p=0.875), respectively.

**Table 2:** Associations between Cabrol shunt and operative mortality in the crude analysis, multivariable analysis and propensity score analyses

Analysis	Operative mortality	P value
No. of events/no. of patients at risk (%)		
Non-Cabrol shunt	71/1082 (6.6)	
Cabrol shunt	146/2201 (6.6)	
Crude analysis—OR (95% CI)	1.012 (0.754 to 1.357)	0.938
Multivariable analysis*	0.957 (0.704 to 1.301)	0.780
Propensity score analysis*		
Adjusted for propensity score*	0.963 (0.714 to 1.299)	0.805
With propensity score matching*	1.073 (0.673 to 1.712)	0.767
With inverse probability weighting*	1.012 (0.749 to 1.368)	0.937
Multivariable analysis†	1.104 (0.765 to 1.592)	0.598
Propensity score analysis†		
Adjusted for propensity score†	1.016 (0.732 to 1.409)	0.925
With propensity score matching†	0.831 (0.479 to 1.442)	0.511
With inverse probability weighting†	0.958 (0.652 to 1.408)	0.827
Multivariable analysis‡	0.990 (0.682 to 1.437)	0.957
Propensity score analysis‡		
Adjusted for propensity score‡	1.020 (0.734 to 1.418)	0.904
With propensity score matching‡	0.663 (0.361 to 1.219)	0.186
With inverse probability weighting‡	0.968 (0.644 to 1.454)	0.875

*Adjustment for coagulation-related parameters (platelet, D-dimer, haemoglobin, international normalised ratio and activated partial thromboplastin time).

†Adjustment for age, sex, body mass index, cardiovascular risk factors and laboratory profiles.

‡Adjustment for age, sex, body mass index, cardiovascular risk factors and laboratory profiles, as well as coagulation-related parameters.

### Subgroup analysis of root procedure

**Figure 2:**
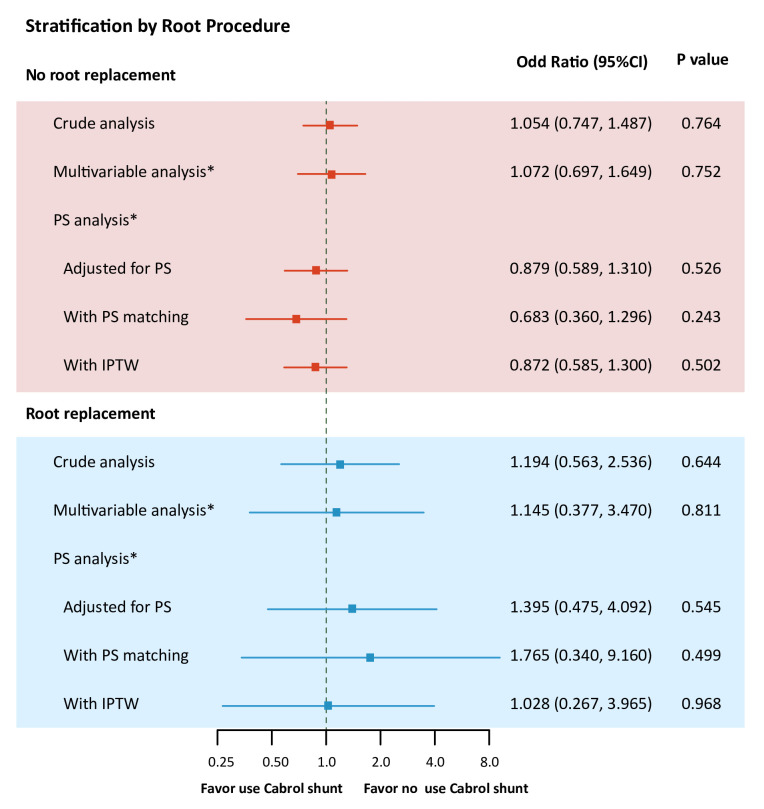
Subgroup analysis of association between Cabrol shunt and primary outcome. (A) Subgroup analysis by root replacement. *Adjustment for age, sex, body mass index, cardiovascular risk factors and laboratory profiles, as well as coagulation-related parameters (platelet, D-dimer, haemoglobin, international normalised ratio and activated partial thromboplastin time). IPTW, inverse probability of treatment weighting; PS, propensity score.

The rate of aortic root replacement was 14.2% (154/1082) in the non-Cabrol shunt group and 50.3% (1107/2201) in the Cabrol shunt group (p<0.001). Stratified by root procedure, patients with Cabrol shunt were likely to have similar risk of operative mortality compared with those without in patients without root replacement (OR 1.054 (0.747 to 1.487); p=0.764) and in patients with root replacement (OR 1.194 (0.563 to 2.536); p=0.644) (P interaction=0.765). After additional adjustment for multivariable and PS analysis, the results were still similar (figure 2; online supplemental table S1).

10.1136/openhrt-2023-002465.supp1 Supplementary data

### Number needed to treat or harm analysis

**Figure 3:**
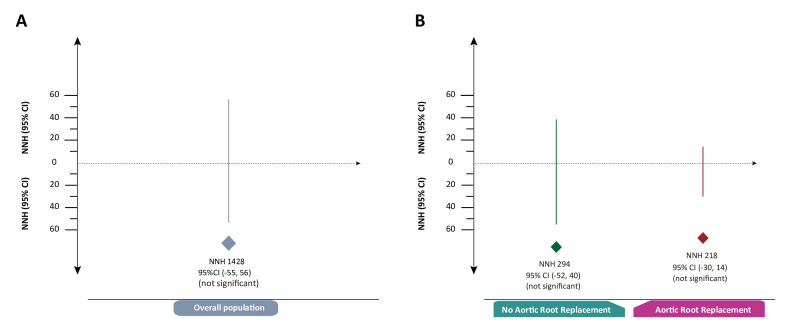
Number needed to treat or harm analysis. (A) Overall population; (B) subgroup by root replacement. NNH, number needed to harm; NNT, number needed to treat; CI=CI interval.

Overall, the number needed to harm was 1428 patients (95% CI −55 to 56; no significant) receiving Cabrol shunt for one patient to cause operative death compared without receipt of Cabrol shunt. Figure 3 showed the number needed to treat or harm analysis across the subgroup within root procedure (with or without root replacement).

## Discussion

In this large sample of TAAD population from China 5A study, we investigated the contemporary use and outcome of Cabrol shunt in TAAD. Overall, no significances were observed between non-Cabrol and Cabrol shunt regarding operative mortality. By subgroup analysis, Cabrol shunt was likely to have similar risk of operative mortality to non-Cabrol shunt regardless of aortic root replacement. Results were similar in multiple sensitivity analysis.

Bleeding complications with aortic root replacement have prompted development of techniques by which the graft is wrapped with the remaining native aorta to control bleeding. Cabrol and colleagues extended this idea by creating a fistula between the periprosthetic space and the right atrium to prevent development of a haematoma under tension with eventual tearing of the aortic wall.^4 7^ Cabrol shunt and its modifications has evolved over the years as an important contribution to controlling haemorrhage in surgical repair of aortic dissection. Much of the early enthusiasm for Cabrol shunt focused on an expected advantage in patients with complex root reconstructions, secondary thoracotomy, coagulation disorders or long-term treatment of aspirin or a preoperative thrombolysis for a presumed myocardial infarction.^24^ In the last decades several technical, materials and pharmacological improvements allowed application of the open technique in TAAD, including zero porosity grafts, tissue glue and polytetrafluoroethylene felts.^25 26^ Given more surgeons abandoned the inclusion technique in favour of the open technique of dissection repair, the application of Cabrol shunt is increasingly rare in surgical repair of TAAD.

Inconsistent with some previous studies,^5–9^ our findings showed the associations observed between Cabrol shunt and operative mortality were not significant across different population subgroups (ie, absence or presence of aortic root replacement), although risk varies to differing extents. Our results suggested that patients with Cabrol shunt did not benefit or harm more than those without Cabrol shunt, regardless of root replacement. These results were similar in multiple sensitivity analysis. It might be explained by the fact that those patients who underwent Cabrol shunt in previous studies were at high risk of excessive bleeding, uncontrollable bleeding, coagulation disorders, complex root procedures or reoperation and Cabrol shunt were applied mainly for the purpose of prophylactic preference.^9 24 27 28^

On the contrary, these patients induced in our study are non-selected, regardless of risk stratifications and Cabrol shunt were applied for different purposes, including prophylactic, routine or remedial preference. In addition, previous studies began several years before our study, during a time when open-repair devices, techniques and strategies were changing rapidly. The quality of the surgical procedure may affect perioperative clinical outcomes, especially for this emergency surgical repair of TAAD. It is less clear that higher surgical quality would result in better operative outcomes after open repair, but it is possible that the steep learning curve for open repair resulted in differences in surgical quality that were reflected in later results. As a result, it makes this procedure seemingly less desirable for use in aortic surgery, especially in root replacement.

Our study has important clinical implications. Although our study showed no statistical difference between the non-Cabrol and Cabrol groups, it does not mean that the Cabrol procedure lost clinical application value in aortic surgery, mainly due to the lack of further subgroup analysis in terms of use purpose to the great extent. A recent study from Elefteriades and colleagues investigated its effectiveness in controlling bleeding, persistent left-to-right shunt, anastomotic pseudoaneurysm and operative, hospital and long-term survival.^29^ In their study, recalcitrant bleeding was successfully controlled by the Cabrol fistula in all 17 patients, and only 2 patients required re-exploration for clot evacuation. These patients all survived to discharge, except for three cases consisting of two deaths from multiple organ failure and one death from intraoperative technical difficulties. In no case did echocardiography (ECHO) identify sustained left-to-right shunting or any patent shunt beyond a few days postoperatively and later CT scan identify continued flow via the Cabrol shunt or any evidence of anastomotic pseudoaneurysm, showing extremely favourable experience for control of intractable intraoperative bleeding in aortic surgery. Similarly positive outcomes have been found in other centres as well.^24 30^ Therefore, it emphasises the importance and necessity of determining the exact indications for previous Cabrol shunt use, especially in case of intractable intraoperative bleeding in some complex, extensive or emergent procedures compared with the conventional method of packing an open chest.

### Limitation

Our study is not devoid of limitations. First of all, due to the lack of sufficient and precise information about the purpose of this method for each patient in which Cabrol shunt was primarily used for the one of the following purposes: prophylactic, routine or remedial, our findings could still be affected by a residual treatment selection bias despite multivariate adjustment and PS analysis. Second, our database does not provide complete information regarding the incidence rate of pseudoaneurysm formation, which might have an important effect on the interpretation of these current results. Finally, the 5A does not contain information on long-term survival outcomes and quality of life. Adverse outcomes might lead to a decreased quality of life and thus, mitigate the beneficial effect on operative mortality resulting in fewer quality-adjusted life years.

## Conclusion

Relying on this large sample study, we found that Cabrol shunt was not associated with either a greatly lowered or an increased risk of operative mortality. This is consistently constant in each subgroup with regard to root replacement. These results suggested that patients did not benefit more from Cabrol shunt with regards to operative mortality. Our study did not support the use of the Cabrol shunt as a routine preventive strategy in the treatment of TAAD.

## Data Availability

All data relevant to the study are included in the article or uploaded as supplementary information.
