# Supplementary material for: Contemporary use and outcome of Cabrol shunt in type A aortic dissection surgery: insight from China 5A study
Source: Open Heart. 2023 Dec 9;10(2):e002465. doi: 10.1136/openhrt-2023-002465 (PMC10729034; doi:10.1136/openhrt-2023-002465)
Supplement: Supplementary data [file openhrt-2023-002465supp001.pdf]

Supplemental Table 1 Subgroup analysis of Cabrol shunt and operative mortality by root replacement

Analysis	Operative mortality	P value
Root procedure		
Patients without root replacement		
No. of events/no. of patients at risk (%)		0.764
Non-Cabrol shunt	63/928 (6.8%)	
Cabrol shunt	78 /1094 (7.1%)	
Crude analysis — odd ratio (95% CI)	1.054 (0.747, 1.487)	0.764
Multivariable analysis*	1.072 (0.697, 1.649)	0.752
Propensity-score analysis*		
Adjusted for propensity score*	0.879 (0.589, 1.310)	0.526
With propensity score matching*	0.683 (0.360, 1.296)	0.243
With inverse probability weighting*	0.872 (0.585, 1.300)	0.502
Patients with root replacement		
No. of events/no. of patients at risk (%)		0.643
Non-Cabrol shunt	8/154 (5.2%)	
Cabrol shunt	68/1107 (6.1%)	
Crude analysis — odd ratio (95% CI)	1.194 (0.563, 2.536)	0.644
Multivariable analysis*	1.145 (0.377, 3.470)	0.811
Propensity-score analysis*		
Adjusted for propensity score*	1.395 (0.475, 4.092)	0.545
With propensity score matching*	1.765 (0.340, 9.160)	0.499
With inverse probability weighting*	1.028 (0.267, 3.965)	0.968

*Adjustment for age, sex, body-mass index, cardiovascular risk factors, and laboratory profiles, as well as coagulation related parameters.

Investigator-initiated Additive Anti-inflammatory Action for Aortopathy & Arteriopathy (5A) Investigators

Hong Liu MD, PhD, Department of Cardiovascular Surgery, the First Affiliated Hospital of Nanjing Medical University, Nanjing 210029, P.R China;

Sheng Zhao MD, PhD, Department of Cardiovascular Surgery, the First Affiliated Hospital of Nanjing Medical University, Nanjing 210029, P.R China;

Yong-feng Shao, MD, PhD, Department of Cardiovascular Surgery, the First Affiliated Hospital of Nanjing Medical University, Nanjing 210029, P.R China;

Zhi-wei Tang, MD, Department of Cardiovascular Surgery, the First Affiliated Hospital of Nanjing Medical University, Nanjing 210029, P.R China;

Si-chong Qian MD, PhD, Department of Cardiovascular Surgery, Beijing Anzhen Hospital, Capital Medical University, Beijing 100029, P.R China;

Hai-yang Li, MD, PhD, Department of Cardiovascular Surgery, Beijing Anzhen Hospital, Capital Medical University, Beijing 100029, P.R China;

Hong-jia Zhang, MD, PhD, Department of Cardiovascular Surgery, Beijing Anzhen Hospital, Capital Medical University, Beijing 100029, P.R China;

Ying-yuan Zhang, MD, Department of Cardiovascular Surgery, the First Affiliated Hospital of Guangzhou Medical University, Guangzhou 510120, P.R China;

Ying Wu MD, Department of Laboratory, the First Affiliated Hospital of Shantou University Medical College, Shantou 515041, P.R China;

Liang Hong MD; Department of Cardiovascular Surgery, Nanjing First Hospital, Nanjing Medical University, Nanjing 210012, P.R China;

Ji-nong Yang MD; Department of Cardiovascular Surgery, the Affiliated Hospital of Qingdao University, Qingdao 266003, P.R China;

Ji-sheng Zhong MD; Department of Cardiovascular Surgery, Xiamen Cardiovascular Hospital, Xiamen University, Xiamen 361004, P.R China;

Tian Niu, MD, Department of Cardiovascular Surgery, Xiamen Cardiovascular Hospital, Xiamen University, Xiamen 361004, P.R China;

Yu-qi Wang MD; Department of Cardiovascular Surgery, Teda International Cardiovascular Hospital, Chinese Academy of Medical Sciences, Tianjin 300457, P.R China;

Bing-qi Sun MD; Department of Cardiovascular Surgery, Teda International Cardiovascular Hospital, Chinese Academy of Medical Sciences, Tianjin 300457, P.R China;

Dong Kai Wu MD, PhD, Department of Cardiovascular Surgery, Xiangya Hospital, Central South University, Changsha 410008, P.R China;

Guo-liang Fan MD, Department of Cardiovascular Surgery, Shanghai East Hospital, Tongji University, Shanghai 200120, P.R China.

Jun-quan Chen, MD, PhD, Department of Cardiovascular Surgery, Tianjin Chest Hospital, Tianjin Medical University, Tianjin 300222, P.R China;

Dong-dong Wu MD, PhD, Department of Cardiovascular Surgery, Beijing Fuwai Hospital, Peking Union Medical College & Chinese Academy of Medical Sciences, Beijing 100037, P.R China;

Yi-yao Jiang, MD, PhD, Department of Cardiovascular Surgery, the First Affiliated Hospital of Bengbu Medical College, Bengbu 233099, P.R China;

Sheng-qiang Zhang, MD, Department of Cardiovascular Surgery, the First Affiliated Hospital of Bengbu Medical College, Bengbu 233099, P.R China;

Si-qiang Zheng, MD, PhD, Department of Thoracic Surgery, Shanghai Lung Hospital, Tongji University, Shanghai 200433, P.R China;

Xin-ya Li, MD, PhD, Department of Cardiovascular Surgery, the First Hospital of University of Science and Technology of China, Hefei 230002, China;

Hong-hua Yue, MD, PhD, Department of Cardiovascular Surgery, West China School of Medicine and West China Hospital, Sichuan University, Chengdu 610041, China;

Zhi-hua Zeng, MD, Department of Cardiovascular Surgery, the Second Affiliated Hospital of Nanchang University, Nanchang 330008, P.R China;

Lu Han, MD, Department of Cardiovascular Surgery, Beijing Chaoyang Hospital, Capital Medical University, Beijing 100043, P.R China.
